# Failure Analysis in Magnetic Tunnel Junction Nanopillar with Interfacial Perpendicular Magnetic Anisotropy

**DOI:** 10.3390/ma9010041

**Published:** 2016-01-12

**Authors:** Weisheng Zhao, Xiaoxuan Zhao, Boyu Zhang, Kaihua Cao, Lezhi Wang, Wang Kang, Qian Shi, Mengxing Wang, Yu Zhang, You Wang, Shouzhong Peng, Jacques-Olivier Klein, Lirida Alves de Barros Naviner, Dafine Ravelosona

**Affiliations:** 1 School of Electrical and Information Engineering, Beihang University, Beijing 100191, China; xiaoxuan.zhao@buaa.edu.cn (X.Z.); boyu.zhang@buaa.edu.cn (B.Z.); kaihua.cao@buaa.edu.cn (K.C.); buaawlz@buaa.edu.cn (L.W.); wang.kang@buaa.edu.cn (W.K.); qian.shi@buaa.edu.cn (Q.S.); wangmx_0615@buaa.edu.cn (M.W.); yu_zhang@buaa.edu.cn (Y.Z.); shouzhong.peng@buaa.edu.cn (S.P.); 2 Spintronics Interdisciplinary Center, Beihang University, Beijing 100191, China; 3 Institut d’Electronique Fondamentale, CNRS UMR 8622, University of Paris-Sud, 91405 Orsay, France; you.wang@telecom-paristech.fr (Y.W.); Jacques-olivier.klein@u-psud.fr (J.-O.K.); dafine.ravelosona@u-psud.fr (D.R.); 4 Laboratoire Traitement et Communication de l’Information, Institut MINES-TELECOM, TELECOM ParisTech, Paris 75634, France; lirida.naviner@telecom-paristech.fr

**Keywords:** magnetic tunnel junction, interfacial perpendicular magnetic anisotropy, process variation, stochastic behavior, barrier breakdown, STT-MRAM

## Abstract

Magnetic tunnel junction nanopillar with interfacial perpendicular magnetic anisotropy (PMA-MTJ) becomes a promising candidate to build up spin transfer torque magnetic random access memory (STT-MRAM) for the next generation of non-volatile memory as it features low spin transfer switching current, fast speed, high scalability, and easy integration into conventional complementary metal oxide semiconductor (CMOS) circuits. However, this device suffers from a number of failure issues, such as large process variation and tunneling barrier breakdown. The large process variation is an intrinsic issue for PMA-MTJ as it is based on the interfacial effects between ultra-thin films with few layers of atoms; the tunneling barrier breakdown is due to the requirement of an ultra-thin tunneling barrier (e.g., <1 nm) to reduce the resistance area for the spin transfer torque switching in the nanopillar. These failure issues limit the research and development of STT-MRAM to widely achieve commercial products. In this paper, we give a full analysis of failure mechanisms for PMA-MTJ and present some eventual solutions from device fabrication to system level integration to optimize the failure issues.

## 1. Introduction

Continuous scaling down of the complementary metal oxide semiconductor (CMOS)technology node drives high power issues due to the increasing leakage currents [1] and large data traffic [2]. To overcome these power issues, non-volatile computing memory devices have received much attention in academic and industrial research [2,3,4,5]. Magnetic tunnel junction (MTJ) switched by a spin transfer torque (STT) mechanism for spin transfer torque magnetic random access memory (STT-MRAM) is considered as the most promising technology [6,7] thanks to its fast speed, infinite endurance, and higher density than conventional computing memory like static random access memory (SRAM). The first generation of STT-MRAM is based on in-plane magnetic anisotropy, which needs the shape of MTJ nanopillar to be in ellipse or rectangular shape to obtain a high thermal energy barrier for data storage [8,9]. As the energy barrier reduces with the size scaling down, this makes in-plane magnetic anisotropy impossible for non-volatile data storage (e.g., >10 years). In addition, its switching current density is dominated by the demagnetization field *H_d_* (see Equation(1)), which is much larger than the magnetic anisotropy *H_k_*; this limits its interest for low power applications. MTJ with perpendicular interfacial magnetic anisotropy (PMA-MTJ) was discovered in 2010 [10,11], which combines a number of advantages, such as high tunnel magneto-resistance ratio (TMR), strong energy barrier for non-volatile data storage, and circular shape, *etc.* As its switching current density depends on the anisotropy *H_K_* not *H_d_* (see Equations (1) and (2), which is much smaller than that in in-plane MTJ, it is promising for low power applications, such as STT-MRAM and all-spin logic device [12,13]:

(1) $$J_{\mathrm{c0\_Inplane}}=\alpha \frac{\gamma e}{\mu_{B}g}(\mu_{0}M_{s})(H_{\mathrm{ext}}+H_{K}+H_{d}/2)t_{\mathrm{sl}}$$

(2) $$J_{\mathrm{c0\_PMA}}=\alpha \frac{\gamma e}{\mu_{B}g}(\mu_{0}M_{s})H_{K}t_{\mathrm{sl}}$$

where α is the magnetic damping constant, γ is the gyromagnetic ratio, *e* is the elementary charge, μ*_B_* the Bohr magneton, *t_sl_* the thickness of the free layer and *k_B_* the Boltzmann constant. µ_0_*M_s_* is the saturation field in the storage layer, *H_ext_* the external magnetic field, *H_K_* the magnetic anisotropy, and *H_d_* the out-of-plane demagnetization field.

**Figure 1:**
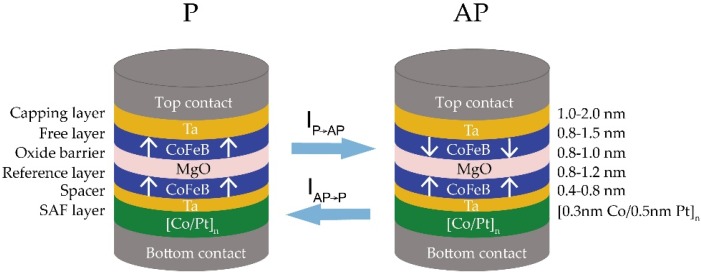
Magnetic tunnel junction with interfacial perpendicular magnetic anisotropy (PMA-MTJ) consists of several ultra-thin layers: two ferromagnetic layers separated by an oxide barrier. Two heavy metal layers are associated with the two ferromagnetic layers, while the synthetic antiferromagnetic (SAF) layer is inserted adjacent the reference layer and bottom electrode. With the spin transfer torque mechanism, PMA-MTJ changes between two states when a bidirectional current *I* is higher than the critical current *I_c_*_0_.

A PMA-MTJ is mainly composed of several ultra-thin layers with a few atom layers: an oxide barrier sandwiched by two ferromagnetic layers, which are associated with two heavy metal layers [10,11]. In addition, synthetic antiferromagnetic (SAF) pinned layers are commonly included into PMA-MTJ by using periodic Co(0.3)/Pt(0.5) ultra-thin multilayers, in order to reduce the offset field, as well as enhance the thermal stability [14]. The interfacial PMA comes from the two interfaces of ferromagnetic layer, for the interface CoFeB/MgO, the origin of PMA is attributed to the hybridization between the iron 3d and oxygen 2p orbitals [15]; for the interfacial CoFeB/Ta, the origin of PMA is attributed to the hybridization between the cobalt 3d and Ta 5d orbitals [16]. Figure 1 demonstrates the main structure of a PMA-MTJ switched by the spin transfer torque (STT) mechanism. Based on the tunnel magneto-resistance effect, the resistance of the nanopillar (*R*_P_ or *R*_AP_) is determined by the corresponding relative magnetization orientation of the two ferromagnetic layers, *i.e.*, parallel (P) or antiparallel (AP) [6]. The resistance difference is characterized by the parameter tunnel magneto-resistance ratio TMR = (*R*_AP_ − *R*_P_)/*R*_P_. Therefore, MTJ can be used to constitute logic “0” and “1” by different configurations.

Despite its excellent potential for non-volatile computing memory, the PMA-MTJ devices suffer from considerable failure issues [17,18,19]. As shown in Figure 1, the interfacial PMA needs the interaction between ultra-thin films with a few layers of atom, which may induce large process variation even with the most advanced process tools for deposition, annealing, and etching. This will cause circuit functional failures due to the deviations of oxide barrier thickness (*t_ox_*), free layer thickness (*t_sl_*) and TMR ratio. The promise of PMA-MTJ for high-density memory needs the nanopillar size smaller than 40 nm [20,21], for this purpose, the oxide barrier should be as thin as possible to reduce the resistance area product (RA) of PMA-MTJ. This will drive serious time-dependent dielectric breakdown (TDDB) failures considering the process variation [22,23]. These challenges are limiting STT-MRAM and all-spin logic devices from research and development to apply widely to commercial products and attract significant research efforts from both academics and industries.

In this paper, we first present the origin of related failures of PMA-MTJ for STT-MRAM use and then propose some eventual solutions based on the analysis. The content will be organized as follows: in the next section, we will analyze the failure issues related to the nanofabrication of PMA-MTJ including device deposition, annealing for material crystallization and nanopillar etching; in Section 3, TDDB failures will be analyzed and at last we propose some solutions to tolerate the failures from the circuit and system functional errors.

## 2. Failure Issues due to Nanofabrication of Magnetic Tunnel Junction Nanopillar with Perpendicular Magnetic Anisotropy

**Figure 2:**
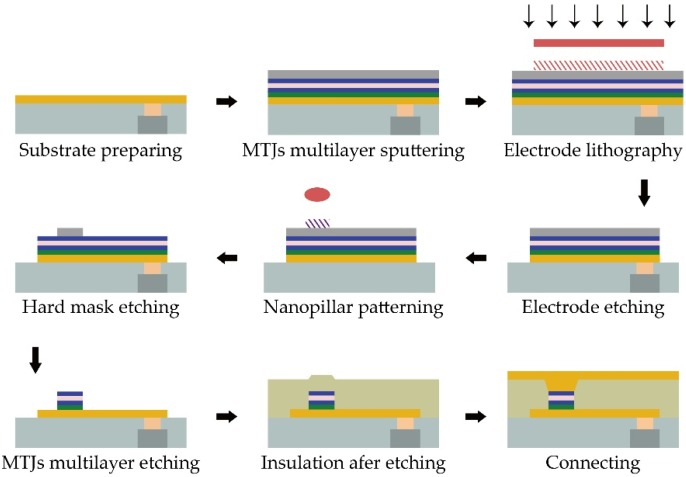
Typical flow of magnetic tunnel junction (MTJ) device fabrication, which mainly comprises stack deposition, patterning, etching dielectric encapsulation, and connecting.

The nanofabrication of PMA-MTJ is based on standard back-end CMOS technology, but it needs additional specific processes. For example, we need the growth of ultra-thin multilayers with a high quality tunnel barrier and precise crystallization matching of ferromagnetic layers to obtain giant TMR ratios and strong PMA. For this purpose, an ultra-high resolution sputtering machine is required. If the process resolution cannot meet the requirements, the large distribution of magnetic and electrical properties may occur, which will lead to poor performance of PMA-MTJ nanopillars. Figure 2 depicts the typical MTJ device fabrication process.

### 2.1. Failure Issues Caused by Deposition Variation

In the process of PMA-MTJ deposition, magnetron sputtering is one of the most advanced tools, which is commonly used in industry, as it provides high growth rate, relatively good yield, and precise thickness control over 300 mm wafer [24]. However, PMA-MTJ based on interfacial effects still suffers from significant failure issues due to the variation of thickness and uniformity under 1 nm or with a few layers of atoms.

Compared with in-plane magnetic anisotropy based MTJ, PMA-MTJ with interfacial magnetic anisotropy is more sensitive to the thickness variation, as it comes from the hybridization of atoms in the two interfaces MgO/CoFeB/Capping layer [15,16]. Both experiments and first-principles calculations have shown that the production of interfacial PMA matters with a certain thickness of ferromagnetic film and capping layer, which is usually a few atoms [10,16,25,26,27]. For instance, in order to trigger a MTJ’s easy axis from in-plane to out-of-plane direction, thinner ferromagnetic film, *i.e.*, less than 1.5 nm in the case of CoFeB/MgO structure, should be deposited [10]. In addition, other magnetic properties, including the offset field and thermal budgets, could be tunable by adjusting the relevant thickness of the individual layers in synthetic antiferromagnetic (SAF) structure, which is mainly because a thickness-dependent co-tuning of exchange coupling of the SAF [28,29].

**Figure 3:**
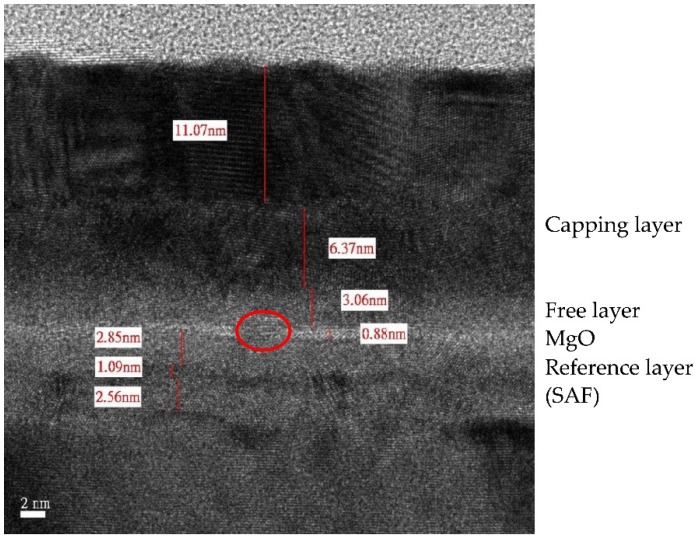
Cross-section image of MTJ stack by transmission electron microscope (TEM), which contains free and synthetic antiferromagnetic (SAF) reference layers separated by ultra-thin 0.88 nm MgO tunnel barrier. This sample was prepared by Anelva HC7100 sputtering equipment. A pinhole exists in the ultra-thinoxide barrier due to rough deposition of MgO, indicated by the red circle.

**Figure 4:**
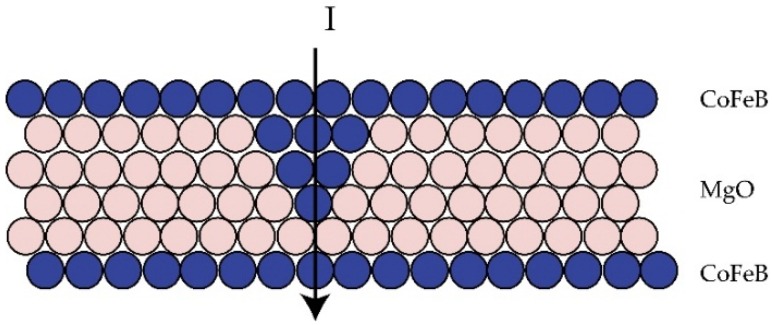
Schematic diagram of the generation of a pinhole. It originates from the rough MgO layer, formed by CoFeB deposition upon defective MgO. The existence of pinholes shunts the current, resulting in the degradation of tunnel magneto-resistance ratio (TMR), and may even cause breakdown.

During the deposition process, uniformity or surface roughness is another critical parameter requiring optimization. The uniformity of ±2% could be obtained by commercialized sputtering system [30]. As shown in Figure 3, the MTJ stack, whose free and SAF reference layers separated by an ultra-thin 0.88 nm MgO tunnel barrier, was deposited by Anelva HC7100 sputtering equipment (Canon, Kawasaki, Japan). In this structure, a high resolution of roughness can be recognized clearly: a pinhole (indicated by the red circle), which is a high-conductance path between two ferromagnetic layers though the oxide barrier, is formed [31]. Figure 4 illustrates that the subsequent CoFeB particles fill in the concave of rough MgO barrier, allowing current go through the metallic contact rather than the barrier, resulting in the degradation of TMR. Thus, unexpected switching may occur during both the writing and reading operations. For the nanoscale MTJ, of which the dimension is quite comparable to that of pinhole, the existence of pinholes could also cause breakdown of MTJ barrier [32], which will be discussed in Section 3 in detail.

In order to control the thickness variation and the uniformity of ultra-thin films, argon pressure, target power, and target to sample distance should be optimized [33]. Experiments exhibited that Ar pressure during sputtering plays a significant role in obtaining high-quality ulta-thin films. When pressure is maintained around 4 mTorr during MgO sputtering, the highest intensity peak appeared in an *X*-ray diffraction (XRD) spectra, which means a promising single-crystallization of MgO [34]. Relatively low Ar pressure helps to avoid the scattering of ejected target materials and, consequently, reduces roughness and thickness variation. However, exceedingly low argon pressure reduces the ionization probability, as well as the deposition rate. Thus, it is essential to balance the argon pressure for reducing the thickness variation of ultra-thin films to avoid the failure of MTJ devices. Previous studies have emphasized the interfaces between CoFeB and MgO play an important role in PMA-MTJ [10]. In addition to the deposition parameters mentioned above, reactive sputtering of the Mg target with an O_2_ atmosphere is another approach to get a good crystallization lattice match between CoFeB and (001) MgO texture [35,36].

### 2.2. Failure Issues Caused by Annealing Parameters

Following the deposition of ultra-thin films, annealing treatment will be applied to obtain perfect lattice structure. In this process, *in situ* annealing using rapid thermal annealing (RTA) method without magnetic field, is often used to obtain a crystalline (001)-oriented MgO tunneling barrier [37,38]. Subsequently *ex situ* annealing (or post-deposition annealing) at temperature *T_ex_* 250–450 °C under magnetic field *H* in a vacuum chamber will be implemented to get better crystallization [39] in both CoFeB layer and MgO barrier.

The magnetic characteristics as well as electrical properties of MTJ nanopillars are strongly influenced by the process variation of *ex situ* annealing treatments. Previous work has demonstrated that the performance of MTJ improves monotonically while starting to increase the annealing parameters (such as *T_ex_*, *H* or annealing times). At certain condition, the best performance could be achieved, then decays when exceeding the optimum parameters. Hence, we can divide annealing treatments into three stages: insufficient annealing, optimum annealing, and over-annealing. However, the optimum annealing paramet ers to get the best magnetic characteristics and the electrical properties do not coincide at the same time.

**Figure 5:**
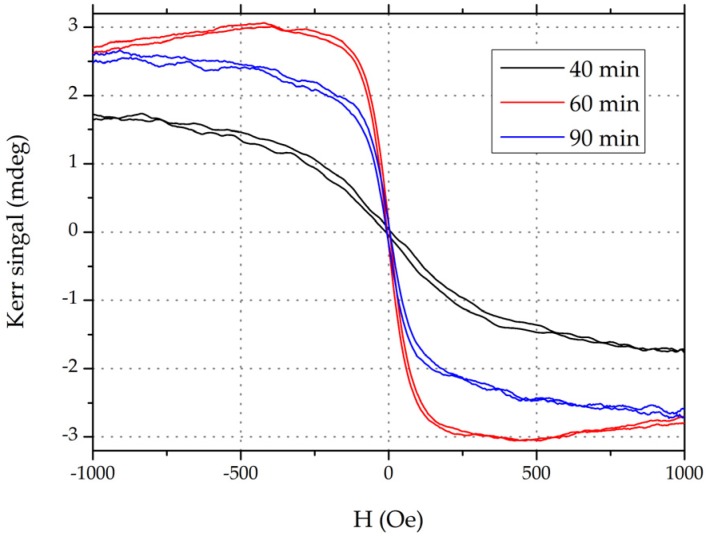
Magnetic curves (measured by NanoMOKE) of MTJ stacks annealed at different annealing times. The film stack of substrate/Ta(3)/MgO(1)/CoFeB(1.1)/Ta(1.5)/Ru(5)/Ta(5) (units in nm) deposited by magnetic sputtering processing are *ex situ* annealed at 300 °C for different annealing times (40, 60 and 90 min) with perpendicular *H* = 0.775 T in a high vacuum chamber.

Since annealing process improves crystallization as well as the interface of ultra-thin films, an *ex situ* annealing process with a certain annealing temperature over a period of time is implemented to enhance PMA [40]. As the magnetic curves shown in Figure 5, reasonable annealing time (60 min, red curve) produced higher *M_S_* and lower *H_sat_*, which means stronger perpendicular magnetization in a typical PMA-MTJ structure of substrate/Ta/MgO/CoFeB/Ta. The up-trend of performance (40 to 60 min) is ascribed to the B absorption by Ta capping layer, leading to a higher *K_eff_* (the effective anisotropy energy density), while the decrease in the case of over-annealing (90 min) is due to the formation of additional magnetic dead layer and intermixing at the Ta/CoFeB interface [41].

With regard to TMR ratio, it is dominated by the coherent tunneling ∆_1_ states of both the MgO tunnel barrier and CoFeB ferromagnetic layers, which gives rise to higher tunneling spin polarization (TSP) [42]. Therefore, it is critical to crystalize the amorphous CoFeB into bcc (001) texture with (001) MgO as template during *ex situ* [43]. However, the structure of CoFeB adjacent to MgO barrier is observed changing from bcc to boride structure due to over-annealing, resulting in the lattice mismatch between barrier and ferromagnetic layers, as well as the degradation of TMR ratio [44]. In addition, inter-diffusion of elements during *ex situ* annealing also leads to rough interfaces [45], attenuation of magnetic characteristics and TMR ratio decreasing, which would cause serious failure issues that cannot be ignored.

Beyond that, there is an extra thermal treatment when integrating MTJs with standard back-end-of-line (BEOL) CMOS processing. As the appropriate annealing temperature is lower than 400 °C, which is the standard CMOS BEOL temperature, MTJs would be overheating after the BEOL process [46]. To enhance thermal tolerance, simultaneously to keep high TMR ratio and low RA, the optimization on MTJ device structure has attracted lots of attention. Co/Pt multilayer-based synthetic ferromagnetic (SyF) reference layers [47,48] and double CoFeB/MgO interface structure [43,49] have proved effective to get a high TMR ratio above 400 °C required for CMOS BEOL.

### 2.3. Failure Issues Caused by Etching Methods

After magnetic films deposition, annealing and optical lithography process, another tough task is MTJ etching, which has an important influence on the quality of devices. To obtain vertical profiles and high-performance MTJ, advanced etching techniques have received particular attention, among which the typical ones include ion milling (ion beam etching, IBE), reactive ion etching (RIE), and inductively-coupled plasma (ICP) [50,51]. For MTJ etching process, several issues may cause the failure: sidewall redeposition, magnetic layer damage, or corrosion, and critical dimension (CD) control. We categorize the failure of MTJ etching on different etching methods and try to give corresponding solutions.

The IBE technique is used as a versatile technology for patterning almost all materials and it is the mainstream tool for the MTJ fabrication in hard-disk industries. The Ar ion beam can be ionized and accelerated in chamber, and subsequently bombarded onto the surface of thin films energetically, which means no chemical reaction involved to cause magnetic film corrosion. However, it suffers a low selectivity between different materials, which makes the choice of hard mask a critical issue. In addition to its low degree of selectivity, the two main drawbacks of IBE are the redeposition issue and shadowing effect [52,53], which may cause electrical shorts and have a limitation on high-density integrations.

**Figure 6:**
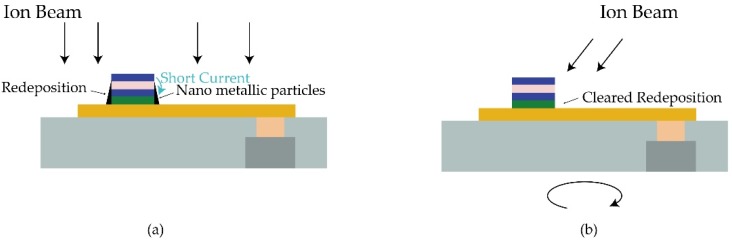
Schematic illustration of (**a**) short-circuit caused by redeposition with no tilt and rotation; (**b**) cleaned sidewall with beam angle and rotation.

**Figure 7:**
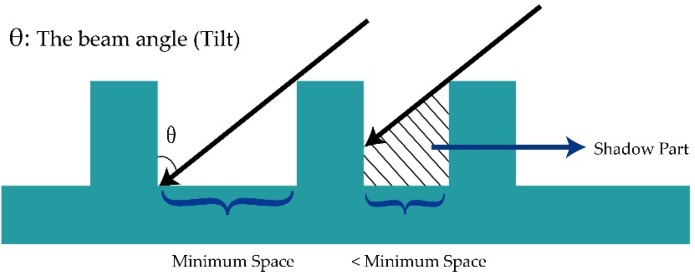
Etching shadow effect with beam angle θ, which is defined as the angle between incident beam and the normal direction of the wafer. The minimum distance between two nanopillar is determined by the height of the pillar and the beam angle.

As shown in Figure 6a, removed atoms diffuse around the pillar and may attach onto its sidewall. In this case, metallic particles on MgO tunnel barrier drives ohmic conduction [54], which may largely decrease the TMR ratio, and even cause a device short failure. Wafer tilt and rotation (Figure 6b) have been introduced to solve this problem [52], whereas the shadow effect consequently happens, as shown in Figure 7. Experiments shown that a 30°–50° wafer-tilt angle make IBE perform better in controlling the profile of nanopillars [55]. In this case, the minimum space between each pillar should arrange from 67 to 138 nm based on the assumption that the height of pillar is 80 nm, limiting its application in high-density array patterning. Meanwhile, low-angle etching for sidewall cleaning has a detrimental effect on controlling the critical dimension. Thus, the angle is a crucial matter to this trade-off dilemma. Chun *et al.*, obtained a nearly vertical PMA-MTJ side profile by implementing a multi-step etching process. In detail, researchers alternated a periodic low-angle (θ = 45°) etching step and higher angle (θ = 60°) etching. The low angle guaranteed a relatively high etching rate while the higher etching angle was used to clean sidewall residues [55].

With respect to reactive ion etching, it is a common method for MTJ etching in semiconductor industries. It provides high throughputs and good selectivity between different materials. Fluorine- or chlorine-containing gases such as chlorine, bromine, and carbonyl are used as the typical reactive gases in RIE, which have high chemical activity. In the RIE process, both physical sputtering and chemical reaction are involved, leading to a relatively high etching rate and good selectivity. However, halogen-based chemicals cause corrosion, due to the non-volatile etching compounds adhering to the ferromagnetic metals [56]. In order to enhance the volatility of the byproducts, processing temperature over 350 °C is implemented [57,58], which is hazardous for high-performance MTJs. DC pulse-biased ICP etching conducted by Yang *et al.* [59] proved to be efficient to reduce the redeposition. By introducing a 60% duty ratio of the DC pulse, decreased residue layer thickness was observed in CoPt/MgO/CoFeB structures compared to that etched with radio frequency continuous wave (RF CW) biasing. This is because during the DC pulse on time, the mono-energetic ions enhance the removal of volatile byproducts produced during the DC pulse off time. In addition, improved etch selectivities of the magnetic layers against the W capping layer were observed with increasing DC bias voltage.

**Figure 8:**
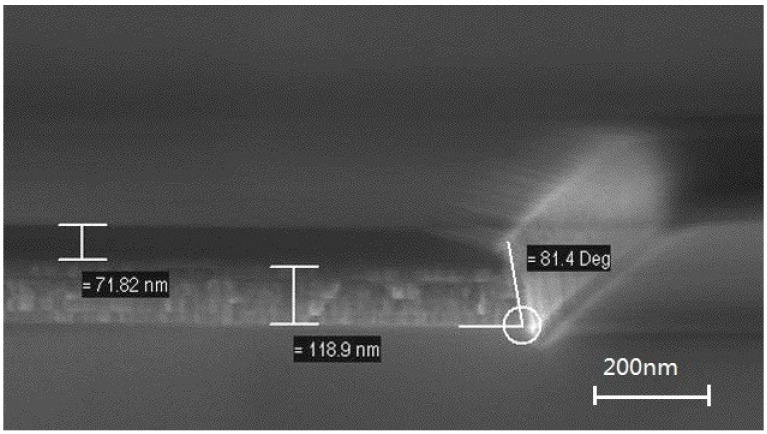
Cross-section image of MTJ stack by scanning electron microscope (SEM), which is etched by inductively-coupled plasma (ICP), shows few redeposition and good device profile.

In the end of 1990s, inductively-coupled plasma (ICP) emerged for adapting a higher aspect ratio and higher etching selectivity in the etching technique, which enhances the density of reactive plasma by adding a top RF source [60,61]. As shown in Figure 8, nearly vertical side profile is observed due to precise control over the plasma’s density and energy. Meanwhile, Me–OH and Ar/Me–OH mixtures have been widely investigated as candidates for their high selectivity against the magnetic metal and hard mask (e.g., Ta/W), combining with non-corrosiveness [62,63]. As the introduction of C–O-based chemistries, ferromagnetic layers could be oxidized during etching, resulting in the degradations of magnetic properties and reduction in TMR ratio [54]. Kinoshita *et al.*, investigated this degradation and proposed post-etching recovery treatment for the CoFeB/MgO-based MTJ by using reductive He/H_2_ plasma to reduce the oxide [64,65]. In their study published in 2014, a TMR ratio of 102% was achieved, which is 5% higher than that of the sample without the He/H_2_ treatment.

Considering the pros and cons of each etching technique, using IBE and RIE-ICP in combination has proven to be effective in both laboratories and industries. In detail, ICP is used to transfer the pattern from electron beam lithography (EBL) photoresist to the hard mask in the first step due to the high reactive selectivities of ICP. Then, multilayers of MTJ should be etched by IBE with different etching angles, followed with some recovery treatments to get better profiles.

## 3. Failure Issues Due to Oxide Barrier Breakdown

**Figure 9:**
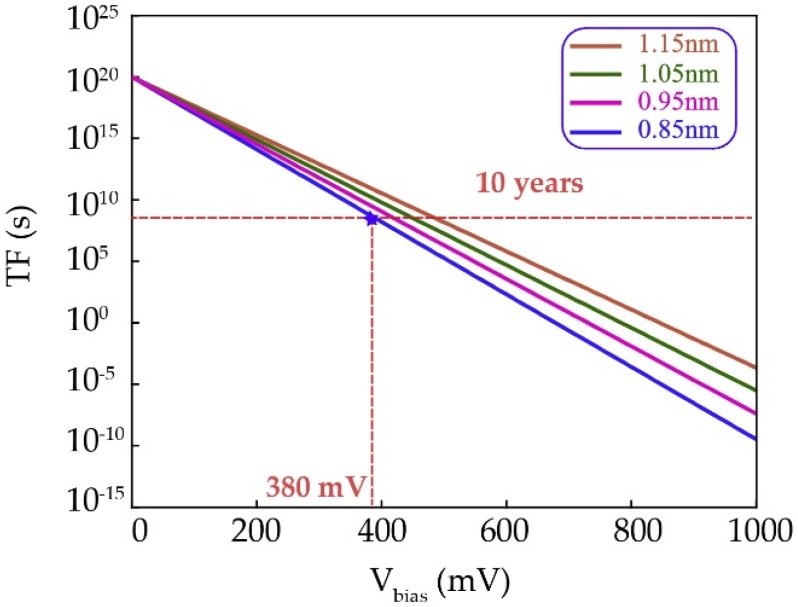
Estimated lifetime of dielectric breakdown *versus* applied bias voltage with different thickness of MgO oxide barrier.

High-speed access is an advantageous merit of STT-MRAM compared to other non-volatile memories based on phase change materials and oxide materials [7]. Recently, a 3.3 ns-access-time was demonstrated in [66], while the write potential *V_w_* was 0.9 V. In this study, a higher access speed comes from a higher *V_w_*, which will threaten the lifetime of devices and cause reliability issues. The widely recognized criterion to determine the MTJ's lifetime and reliability is the time-dependent dielectric breakdown (TDDB), referring to the physical phenomenon where a dielectric, stressed with a constant electric field lower than the breakdown strength (defined as the breakdown voltage when the lifetime is shorter than 10^−10^ s) such as 1.0 V for 0.85 nm MgO as shown in Figure 9 [67], will still breakdown after a certain period of time [68]. In this case, the resistance of a dielectric drops to be more conductive.

In the previous work, two distinct breakdown mechanisms were observed: intrinsic breakdown and extrinsic breakdown [32]. The intrinsic breakdown shows the characteristic that an abrupt decrease resistance occurs when a critical current through the barrier, due to the interaction between the dipole moment of a bond and the applied field [69]. This type of electric breakdown shall be called “hard” breakdown, which could be fitted with the *E*-model. In this model, the lifetime of MTJ could be described in Equation below [70]:

(3) $$\ln\left(TF\right)\propto \frac{\Delta H_{0}}{k_{B}T}-\gamma E_{ox}$$

where *TF* is the time to failure, $\Delta H_{0}$ is the enthalpy of activation, *E_ox_ = V_ox_/t_ox_* is the electric field in the oxide, and γ is the field acceleration parameter. The equation presented above shows that the thickness of oxide barrier has an important effect on the lifetime of MTJ. Figure 9 shows that the lifetime of MTJ decreases by increasing the stress voltage *V_ox_* and decreasing the oxide barrier *t_ox_*. As the lifetime of MTJ is extremely sensitive to the oxide barrier thickness and, hence, any variations of the oxide barrier thickness can have an important impact on the reliability of MTJ.

**Figure 10:**
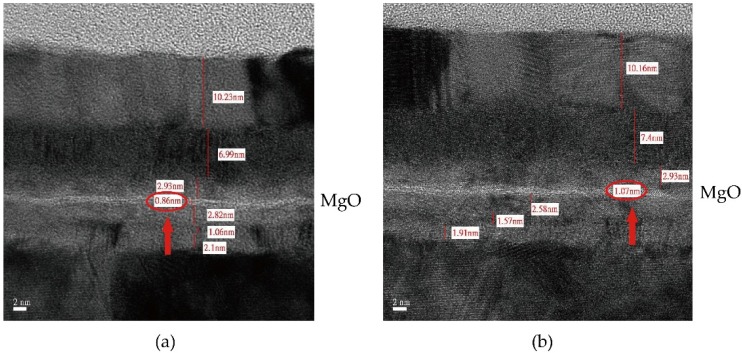
Cross-section image of MTJ stack by transmission electron microscope (TEM), sputtered by Anelva HC7100 sputtering equipment. Multilayers with different ultra-thin MgO oxide barrier thickness: (**a**) 0.86 nm and (**b**) 1.07 nm, respectively, while the nominal thickness is 1 nm.

However, as discussed in Section 2, even the most advanced sputtering system may cause more than 2% nonuniformity during the deposition and this can be accumulated for the multi-layer deposition. As the cross-section images shown in Figure 10, the oxide barrier exhibits different thicknesses for different dies of the same wafer, while the nominal thickness is 1 nm.

In addition to the different TMR ratio and the resistance area product (RA) caused by the variation of oxide barrier thickness, the lifetime of MTJ is also related to it. It is worth noting that the lifetime of MTJ would be significantly overestimated if we do not consider the oxide process variation.

Extrinsic breakdown is another type of breakdown mechanism, also called “soft” breakdown (SBD), which has a slower decline in resistance compared with HBD. Theoretically, the existence of pinholes in the oxide barrier drives a conductance path when an electric current across the MTJ pillar. The ohmic heating around the circumference of a pinhole accelerates its growth until the breakdown occurs [32]. The major difference between SBD and HBD is that the former is related to the barrier imperfection, whereas the latter occurs in a well-formed tunneling barrier. According to the previous TDDB analysis based on the in-plane MTJs, SBD was observed in a few percent of devices and can be nearly neglected. However, the properties of PMA-MTJ is much more sensitive to the interfaces of CoFeB/MgO as the perpendicular magnetic anisotropy here is an intrinsically interfacial effect. It means that the interface quality of the multilayers has important implications for the magnetic properties such as PMA and TMR. Thus, the existence of pinholes becomes a major issue to investigate the breakdown failure mechanism of PMA-MTJ. In addition, annealing process caused TDDB could be classified as SBD. A decreasing breakdown voltage was observed with overannealing temperature (higher than 400 °C), which is mainly because of ferromagnetic material diffusion towards the barrier interface and sacrificing barrier quality [71].

In order to avoid breakdown during STT-MRAM operation, researchers should optimize deposition conditions and the annealing process as mentioned in Section 2. Meanwhile, structure improvement offers a new idea to optimize the breakdown voltage. Hu *et al.*, reported that about 0.7 V *V_breakdown_* was achieved with double tunnel barriers compared to that of 0.2 V with conventional single tunnel barrier devices [72].

## 4. Failure Tolerant Design Techniques

Failure tolerance by manufacturing process aims to reduce the failure rate from fabrication point of view. Unfortunately, we can only optimize it and it is unable to overcome this issue definitively due to the small size of PMA-MTJ nanopillar and its interfacial effects between some layers of atoms. Thereby, failure tolerance at the circuit or system level is required to eliminate the failures and allow the integrated circuits to be functional. This method is currently become a hot topic for the non-volatile memory community, as it is more efficient and cost effective [73].

From the circuit or system design point of view, failures can be classified into two categories based on the physical nature, including soft failure (e.g., wrong signal) and hard failure (e.g., device damage) [17,74,75,76]. The former is mostly related to the environment fluctuations or intrinsic physical mechanisms, like thermal stability, radiation and stochastic switching property. These failures are temporary and can be corrected by a new signal. The latter is mainly caused by the process imperfection (e.g., deposition variation, over annealing and etching, *etc.,* as analyzed above) as well as oxide barrier breakdown (TDDB) [17,23,75]. These failures are persistent and uncorrectable, but generally can be detected (e.g., built-in self-test) after chip fabrication or by online test during usage. Then corresponding circuit or system level techniques (e.g., built-in self-repair) can be employed to tolerate them [77,78]. When employing MTJ in real applications, these failures should be seriously addressed to guarantee the product yield and reliability.

**Figure 11:**
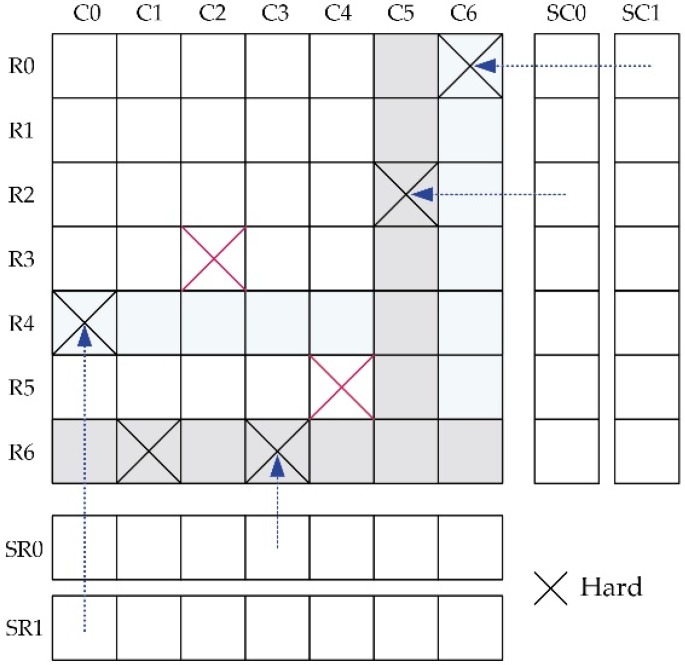
Schematic of the hard failure repair technique with redundancy.

Generally, soft and hard failures are addressed separately in practice. As discussed above, the hard failures are persistent and can be detected. Therefore, we can tolerate these hard failures based on the detection information (failure bit-map). One of the intuitive and direct techniques is to mask the hard failures with redundancy, which means replacing the cells (in hard failures) with good ones [78,79]. As shown in Figure 11 is an example to illustrate the concept. Assume there is a 7 × 7 array with seven hard failures, two redundant rows (SR0 and SR1) and columns (SC0 and SC1). Considering the repair-most algorithm [80], then SR0 is used to replace the R6, SR1 to repair R4, SC0 and SC1 are used to replace C5 and C6, respectively. Unfortunately, two hard failures (cell (R3,C2) and cell (R5,C4)) remain un-repaired after all the redundant rows/columns are consumed, resulting in repair failure. If we want to achieve a 100% repair rate, two additional redundant rows or columns are required. This technique is rather robust to tolerate hard failure; however, one of the most critical problems with this technique is that it is rather redundancy-greedy, leading to huge hardware overhead. To alleviate the hardware overhead issue, many optimization techniques have been proposed. Benso *et al.* [81] proposed to replace only the individual cells (with hard failures) instead of the whole row/column by sacrificing the access complexity. Lu *et al.* [82] proposed a synergistic approach to tradeoff between the hardware overhead and access complexity. The concept is to replace the row/column containing more than two hard failures with a new row/column, but to mask the isolated hard failures by bit flipping.

There are mainly four soft failure sources [17,83,84,85], including write failure due to the intrinsic stochastic STT-driven MTJ switching mechanism, retention failure due to limited thermal stability, radiation effects, and read disturbances due to the large read currents for enough sensing margin. Correspondingly, researchers have proposed techniques to tolerate these failures. For example, Lakys *et al.* [86] and Suzuki *et al.* [87] proposed self-check write circuits to avoid the stochastic STT-driven MTJ switching effect by utilizing a write-verify operation. The concept is that the write circuit performs a read (or verify) operation after each write operation and executes a second write operation if the data stored in the MTJ is different from the intended input one. Kang *et al.* [88] proposed a novel read circuits to address the read disturbance issue by accurately clamping the read current. With a current conveyor, the read current flowing though the MTJ is accurately clamped, thus the read disturbance can be well controlled. However, it should be noted that all these circuit-level design techniques cannot eliminate the soft failures completely. Therefore, system-level design techniques are generally indispensable. As we know, soft failures are temporary and unpredictable. Techniques to tolerate the soft failures should cover all the possibilities.

**Figure 12:**
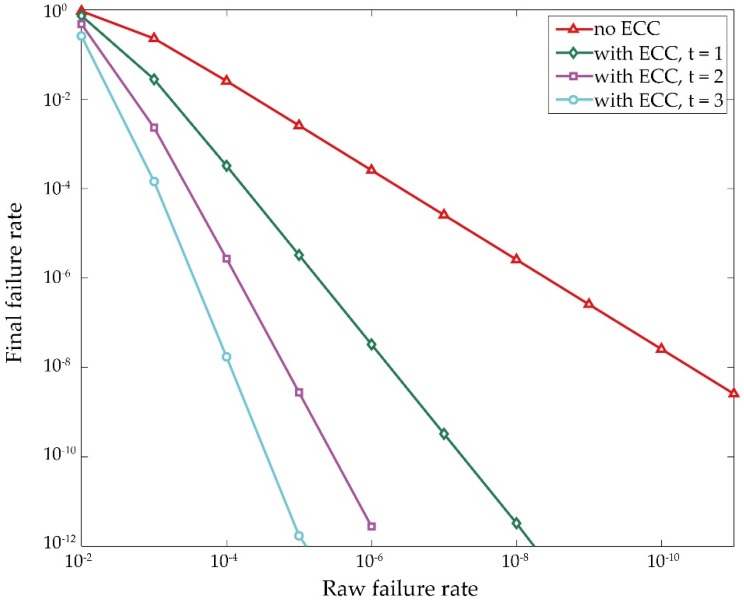
Final failure rate after applying Error correction code (ECC) (with codeword length of 256 bits).

Error correction code (ECC) is one of the most popular system-level techniques [89,90,91] and is rather robust against soft failures by introducing redundant parity check bits. Figure 12 shows the relationship between the raw failure rate and the final failure rate after applying ECC, given a specific codeword size (e.g., 256 bits). Here *t* is the failure correction capability of ECC (it denotes the maximum failures that an ECC correct). As can be seen, the final failure rate decreases dramatically as *t* grows. However, it should be noted that the performance overhead (area and latency) also greatly increases as *t* (or raw failure rate) grows. Therefore, combining circuit- and system-level design techniques is the most preferable strategy for tolerating soft failures, in which circuit level techniques are employed to reduce the raw failure rate while system level techniques are utilized to correct he remaining failures.

## 5. Conclusions

In conclusion, this paper presents a detailed analysis on the failure origin of PMA-MTJ nanopillars. We identified that the interfacial PMA is extremely sensitive to the nanofabrication process and becomes the major cause of process variation, deep sub-micron MTJ nanopillars suffer from the TDDB failure issue due to the low RA requirements and there is no efficient solution to make perfect, large device arrays. Based on this analysis, we proposed a number of methods to reduce the failure rate, from the point of view ultra-thin film deposition. With regard to the introduction of correcting mechanisms at the circuit and system level read speed, as well as capacity, of STT-MRAM must be affected. Hence, the refinement and optimization of multi thin film interaction should be made during fabrication to minimize process variation. This work would help both the academics and industries to understand the critical issues of STT-MRAM behind its great advantages in terms of power, speed, endurance, and non-volatility, *etc.*
